# The Progress in Molecular Transport and Therapeutic Development in Human Blood–Brain Barrier Models in Neurological Disorders

**DOI:** 10.1007/s10571-024-01473-6

**Published:** 2024-04-16

**Authors:** Joanna Korszun-Karbowniczak, Zuzanna Joanna Krysiak, Joanna Saluk, Marcin Niemcewicz, Robert Zdanowski

**Affiliations:** 1 Laboratory of Molecular Oncology and Innovative Therapies, Military Institute of Medicine National Research Institute, 128 Szaserów Street, 04-141 Warsaw, Poland; 2 BioMedChem Doctoral School of the University of Lodz and Lodz Institutes of the Polish Academy of Sciences, 21/23 Matejki Street, 90-237 Lodz, Poland; 3 Department of General Biochemistry, Faculty of Biology and Environmental Protection, Institute of Biochemistry, University of Lodz, 68 Narutowicza Street, 90-136 Lodz, Poland; 4 Biohazard Prevention Centre, Faculty of Biology and Environmental Protection, University of Lodz, 68 Narutowicza Street, 90-136 Lodz, Poland

**Keywords:** Blood-brain barrier (BBB), Hypoxia, BBB permeability, Tight junctions

## Abstract

The blood–brain barrier (BBB) is responsible for maintaining homeostasis within the central nervous system (CNS). Depending on its permeability, certain substances can penetrate the brain, while others are restricted in their passage. Therefore, the knowledge about BBB structure and function is essential for understanding physiological and pathological brain processes. Consequently, the functional models can serve as a key to help reveal this unknown. There are many in vitro models available to study molecular mechanisms that occur in the barrier. Brain endothelial cells grown in culture are commonly used to modeling the BBB. Current BBB platforms include: monolayer platforms, transwell, matrigel, spheroidal, and tissue-on-chip models. In this paper, the BBB structure, molecular characteristic, as well as its dysfunctions as a consequence of aging, neurodegeneration, or under hypoxia and neurotoxic conditions are presented. Furthermore, the current modelling strategies that can be used to study BBB for the purpose of further drugs development that may reach CNS are also described.

## Introduction

Brain injuries after cardiac arrest are recognized by American Heart Association as crucial area in clinical research. Each year almost 800,000 individuals are suffering from new or recurrent stroke (Christophe et al. 2020), while number of seniors and other patients with central nervous system (CNS) diseases are growing. The existing treatment strategies fall behind mainly due to the fact that development of drugs for brain disorders is slow in comparison to other therapeutic areas (Dong 2018).

Blood–brain barrier (BBB) is a delimiter between the blood and CNS (Sivandzade and Cucullo 2018). In vivo*,* BBB is defined by its ability to selectively regulate the permeability for substances to cross from the circulating blood into the brain (Logan et al. 2019). The BBB interface between the CNS and the blood protects against pathogens and toxic compounds as well as transports nutrients to the brain (Sivandzade and Cucullo 2018; Dunton et al. 2021; Pardridge 2022). The BBB major component is endothelial cells (ECs), which are connected with pericytes (PCs), astrocytes (ACs), and neurons. Monolayer of tightly sealed ECs expressing low paracellular and transcellular permeability. ECs form a selective barrier and regulate the substances entry into the brain due to tight junctions, specific molecular transporters, and polarized efflux pumps (Kaisar et al. 2017; Vatine et al. 2019; Dunton et al. 2021).

Several neurological diseases are associated with BBB breakdown. Post-mortem analyses revealed capillary leakages into the brain in Alzheimer’s disease (AD) patients. The proteins derived from the blood were found with Amyloid beta protein (Aβ), the main component associated with theory of AD pathogenesis (Sweeney et al. 2019). Endothelial and pericyte degeneration have been confirmed in AD and other neurodegenerations like Amyotrophic lateral sclerosis (Armulik et al. 2010; Sengillo et al. 2013).

BBB is an impregnable barrier for more than 98% of small molecule drugs that cannot pass the BBB. Therefore, brain drug development studies are based on only 2% of molecules that in a lipid-mediated diffusion manner can penetrate the BBB (Pardridge 2022). To improve transport through the BBB, carrier-mediated transport, receptor-mediated transcytosis, nanoparticles, or focused ultrasound can be used (Pardridge 2015; Yemisci et al. 2015; Burgess and Hynynen 2016; Thrippleton et al. 2019). Dynamic contrast-enhanced MRI stands for BBB permeability assessment in cerebral small vessel disease and other related conditions in clinical (Taheri et al. 2011).

Models of the human BBB allow to study the molecular transport in health and disease conditions (Bell et al. 2010; Kaisar et al. 2017; Hajal et al. 2022). The models development that allow to understand of BBB molecular structure and function can lead to design a solutions that enable the transport of therapeutic agents into the brain (Bergmann et al. 2018). Since barriers differ among animals and humans, BBB studies should be based on human-specific models: the simplest ones, such as Transwell, have a rigid surface that preclude cell–cell interactions, thereupon 3D technology (Vatine et al. 2019). BBB organoids can be considered as a reliable multicellular platform to study brain-penetrating agents, nevertheless, obtaining reproducible spheroids remains a challenge (Cho et al. 2017; Bergmann et al. 2018).

BBB studies can put the spotlight on potential treatment targets (Sengillo et al. 2013). Understanding the processes regulating barrier formation and function drives to development of many in vitro BBB models; patient-specific models would be the future of the modelling (Sivandzade and Cucullo 2018). In vitro models have been developed to mimic in vivo conditions. They comprise static and dynamic platforms built of different cell types, including primary cell lines, immortalized cell lines, and stem cells (Kaisar et al. 2017; Sivandzade and Cucullo 2018). Each of the available BBB models has some limitations, described in this review. They can be used for functional testing, to assess whether certain substances can penetrate or not the BBB.

## The Blood–Brain Barrier Structure

**Fig. 1:**
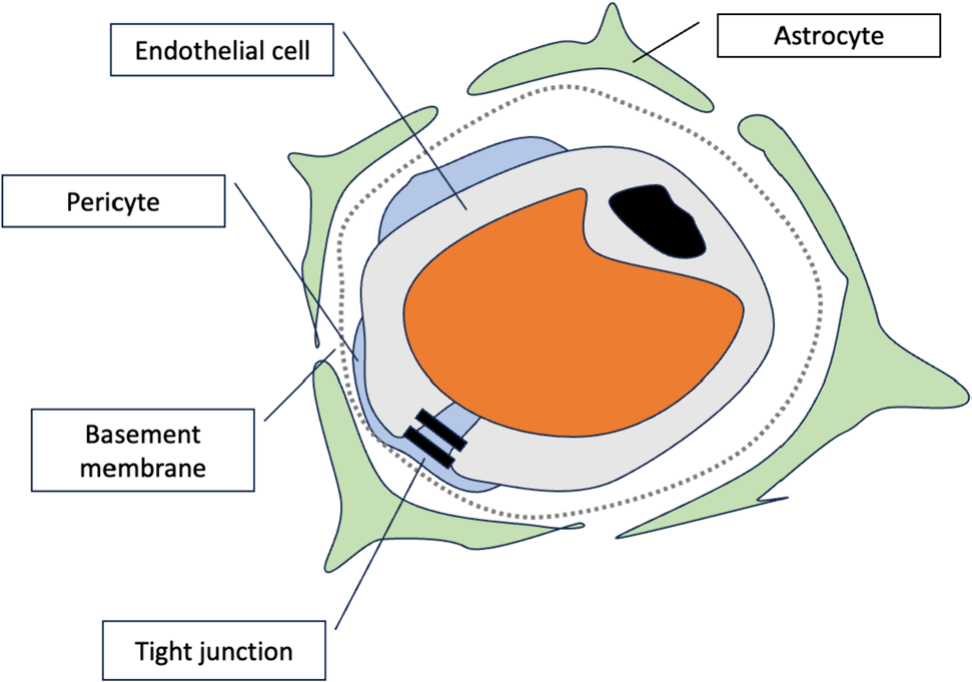
Schematic representation of the BBB; Mammals have a BBB (endothelial barrier) sealed with tight junctions (Dunton et al. 2021)

The ECs are the main cells forming the BBB and are playing the crucial role in BBB functionality. Nevertheless, PCs, ACs, microglia, neuronal cells, and perivascular macrophages affect the ECs activity (Dunton et al. 2021). ECs are sealed with glycocalyx and transporter proteins on the luminal side and coated in the basement membrane, while the ACs, and PCs on the abluminal side (Knox et al. 2022). The BBB outer layer is created mainly by ACs, while the core is formed by ECs (Fig. 1). The PCs are ECs supportive cells, involved in inducing and maintaining BBB properties and integrity (Hladky and Barrand 2016; Jiang et al. 2018). Brain capillary diameter is modulated by PCs through vessel wall constriction, while ACs regulate the contractibility of intracerebral vessels (Peppiatt et al. 2006; Takano et al. 2007; Kuchibhotla et al. 2009). Fibronectin, laminin, collagen, and elastin structural proteins constitute the stable basement membrane (Bagchi et al. 2019). PCs share the basement membrane with ECs and are involved in the regulation of their differentiation, migration, and proliferation (Cardoso et al. 2010).

A neurovascular unit (NVU) is a functional structure built of brain vascular and neural components. There are neural (neurons, microglia, ACs, and oligodendrocytes) and vascular (ECs, PCs, and vascular smooth muscle cells) components of the NVU (Wang et al. 2021). BBB is located in the central part of NVU (Sweeney et al. 2019). Besides NVU cells, there is the non-cellular extracellular matrix (ECM) that conditions cell adhesion, and provides structural support and biochemical signals to the NVU cells (Zidarič et al. 2022).

EC layer is highly polarized. There is a significant difference in the composition of proteins in the luminal and abluminal side, and is metabolically active due to a very high density of mitochondria. The BBB metabolic activity is almost five times higher than in other blood-organ barriers, therefore, brain ECs form a selective diffusion barrier for substances entering the brain (Kaisar et al. 2017).

To maintain the homeostatic balance of the CNS, the transport of substances to and out of the brain has to be strictly regulated (Dunton et al. 2021). BBB cells express the bulk of transporters, efflux pumps, receptors, ion channels, and regulatory molecules (Sweeney et al. 2019). Extreme temperature, increase in inflammatory cytokines (especially IL-6), and reactive oxygen species (ROS) affect the BBB permeability (Bernardo-Castro et al. 2020). The disruption of BBB is attributable to age and can be considered as a hallmark of age-related disorders. Any disturbances in BBB function can lead to: ion dysregulation and neuronal dysfunction and degeneration, invasion of immune cells, toxins, and pathogens to the CNS. Breakdown of the BBB includes: increased leukocyte infiltration, changes in molecular transport, EC shrinkage, and loss of tight junction proteins (TJs) (Dunton et al. 2021; Knox et al. 2022).

Within the endothelial space among ECs, ACs, and PCs there are tight junction proteins, crucial for the barrier function. TJs form a multiprotein cytoplasmic proteins complex (zonula occludens-1 (ZO-1), ZO-2, ZO-3, and cingulin) and transmembrane proteins (junctional adhesion molecules (JAMs), occludin and claudin). The lack of any of these proteins meaningfully affects the BBB integrity and functionality (Zidarič et al. 2022).

## In Vitro BBB Models

### Cell Types

Brain microvascular endothelial cells (BMECs), or other ECs, are supported by basement membrane. Rat brain endothelial cells (RBE4), mouse brain endothelial cells (bEND.3) immortalized cell lines are most frequently involved in BBB in *vitro* studies due to their barrier properties (Lippmann et al. 2013). Besides those two rodent-origin cell lines, BBB hCMEC/d3 is used as a human model of BBB. (Weksler et al. 2013). All mentioned express endothelial markers such as claudin-5, ZO-1 and occludins (Watanabe et al. 2013; Sun et al. 2022). ACs, PCs, and microglia can be used for functional BBB formation (Bagchi et al. 2019). Analysis of molecular permeability constitutes a major application of BBB models (Wu et al. 2021; Hajal et al. 2022). Other applications are the assessment of the physiological and pathological responses to specific stimuli, CNS drug discovery, and drug permeability screening (Kaisar et al. 2017). Modelling of human-specific BBB is crucial as it significantly differs across species (Logan et al. 2019).

Pluripotent stem cells can serve as an experimental model to study brain architecture and neurodegenerative processes. The human cortical organoids (hCOs), three-dimensional, pluripotent stem cell-derived, allow to examine neurological disorders and initial development of the human brain. Developing functional vasculature is essential for neuron progenitor differentiation. Utilization of the human embryonic stem cells (hESCs) leads to the formation of vasculature-like network in hCOs. Human brain organoids transplanted to the mouse’s brain became entwined by murine vessels, and so cell survival and maturation increase (Mansour et al. 2018; Cakir et al. 2019).

### iPSCs

Human-Induced Pluripotent Stem Cells (iPSCc) are generated from reprogrammed somatic cells. Somatic cells usually origin from a blood sample or a skin biopsy, returned to a stem cell-like state by introducing transcription factors: MYC, KLF4, SOX2, and OCT4. iPSCs like other stem cells can differentiate into many cell types. Use of targeted stimulation allows to obtain specific cell types, required for the research. iPSCc are widely used in neurological studies. This model is useful for studying brain structure as well as in drug screening (Wu et al. 2021). It allows to obtain the co-culture of cells required for BBB construct (Logan et al. 2019).

Vatine et al. obtained iPSC-derived brain microvascular endothelial-like cells that with ACs and neurons formed a tight monolayer with specific brain vasculature markers, creating a barrier and protect neural cells from toxins. iPSCs derived from patients with neurological diseases allow to predict a specific lack of transporters and affected barrier integrity. Created NVU allows to study of BBB functions, drug screening, and to model neurological disorders (Vatine et al. 2019). Kadry et al. used iPSCs-derived BMECs and transwell system to examine the effect of smoking and metformin on the BBB. The barrier integrity was assessed by ZO-1, claudin-5, and occludin expression and distribution. The results showed that, the expression of claudin-5 was significantly decreased and the distribution of ZO-1 was altered under harmful conditions (Kadry et al. 2021). Wei et al. differentiated mesenchymal stromal cells from human iPSCs and drugged mice with them. It resulted in improved BBB integrity and decreased inflammation in the mice’s CNS (Wei et al. 2023).

Despite its great potential, applying iPSCc has some limitations. Reprogrammed cell culture is heterogenic, iPSC-derived cells are lack of age-related epigenetics (Wu et al. 2021). Differentiation efficacy is limited as well (Logan et al. 2019). Moreover, most of the studies finally use 2D model with one type of reprogrammed cells, so without cell–cell interactions cannot imitate an in vivo-like environment (Wu et al. 2021).

### Models

2D cell cultures based on extracellular matrix components and one cell type have been used to study cell signaling pathways and cellular responses. Three-dimensional cell cultures involve extracellular matrices, hydrogel cultures, spheroids, and solid scaffolds (Kaisar et al. 2017). 3D models (spheroids) are a way out for a better understanding of the cell interactions among BBB cell types. However, transwell-based modeling systems, brain microvessels, extracellular matrix-based modeling platforms, and microfluidic systems have been used to study BBB as well (Table 1) (Waldau 2019; Wu et al. 2021).

**Table 1:** BBB model types

Model type	Cell types	Application	Limitations	References
2D cell culture	ECs, alternatively ACs, PCs or neurons added to the culture	Functional outcomes of the BBB	Lack of interactions among various BBB cells; higher permeability of 2D BBB model than in vivo	Buzhdygan et al. (2020), Kaisar et al. (2017), Hajal et al. (2022)
Transwell	Monolayer cell culture, cell lines can be seeded on both sides of the membrane with tiny pores to achieve contact co-culture	Transport mechanisms, functional testing, drug screening or genotyping, endothelial integrity, neurotoxicity, disease modeling and organogenesis, long-term effects of drugs, acute and BBB chronic injury	Lack of shear-stress, lack of direct cell–cell contact, low levels of BBB regulatory proteins	Knox et al. (2022), Chung et al. (2022), Rice et al. (2022) Nakagawa et al. (2009), Waldau (2019), Wu et al. (2021)
Matrigel	A platform with PCs and ACs on the ECs abluminal side	Induction of the vascularized human brain organoids, 3D printing and bioprinting, physiological mechanisms, infection experiments	Material may be immunogenic, also poor control of mechanical properties of the scaffold, vascular permeabilities close to the permeability in 2D models	Patel and Alahmad (2016), Simöes Da Gama and Morin-Brureau (2022), Berg et al. (2018), Urich et al. (2013), Waldau (2019), Komura et al. (2015), Kleinman and Martin (2005), Kozlowski et al. (2021), Hajal et al. (2022)
Spheroidal	ECs, ACs and PCs auto-assemble without scaffolding material, alternatively more neural cells can be used: microglia, oligodendrocytes and neurons	The transport of brain penetrating agents and organogenesis, formation of patient-specific BBB models, organogenesis, neurotoxicity, cellular viral infectivity, permeability and function of different drugs, long-term effects of drugs, disease modelling, efflux of the ABCB substrates, imaging of tight-junction and transporter proteins, gene and protein analysis, cell–cell contact and barrier formation	The integrity of spheroid can be tested only once	Knox et al. (2022), Bergmann et al. (2018), Simonneau et al. (2021), Prasad et al. (2021), Campisi et al. (2018), Waldau (2019), Hajal et al. (2022), Bhalerao et al. (2020), Stone et al. (2019), Wu et al. (2021), Eilenberger et al. (2021), Hajal et al. (2022), Tietz and Engelhardt (2015), Nakagawa et al. (2009), Hatherell et al. (2011), Urich et al. (2013), Knox et al. (2022)
Tissue-on-chip		Drug delivery and CNS neurotoxicity studies, cell-to-cell interactions and structures, acute and BBB chronic injury, to create microenvironment for brain organoids development, cell migration due to gradients of chemotactic cues	Smaller cell amount than in other models; model cannot precisely imitate in vivo BBB structure and function	Chung et al. (2022), Wang et al. (2018), Kilic et al. (2016), Kaisar et al. (2017), Wu et al. (2021), Campisi et al. (2018), Aazmi et al. (2022)

*ECs* endothelial cells, *ACs* astrocytes, *PCs* pericytes

### 2D Models

The simplest in vitro BBB models are human 2D monolayer platforms. ECs are seeded on the top of the hydrogel layer (often collagen) or on porous membrane. Sometimes ACs, PCs, or neurons are added to the culture. This method is simple and easy. Nevertheless, limited due to the lack of interactions among various BBB cells and gives a poor prognosis for drug-tissue interactions. Buzhdygan et al. used a 2D and a 3D vessel-like in vitro models to examine the spike protein of SARS-CoV-2 that affects the function of the BBB and compromises its properties. 2D monolayer platforms can be used to test functional outcomes of the BBB, but the properties of the model are limited. Permeability of 2D BBB model is one to three times higher than in vivo ones (Buzhdygan et al. 2020; Hajal et al. 2022).

### Transwell Model

**Fig. 2:**
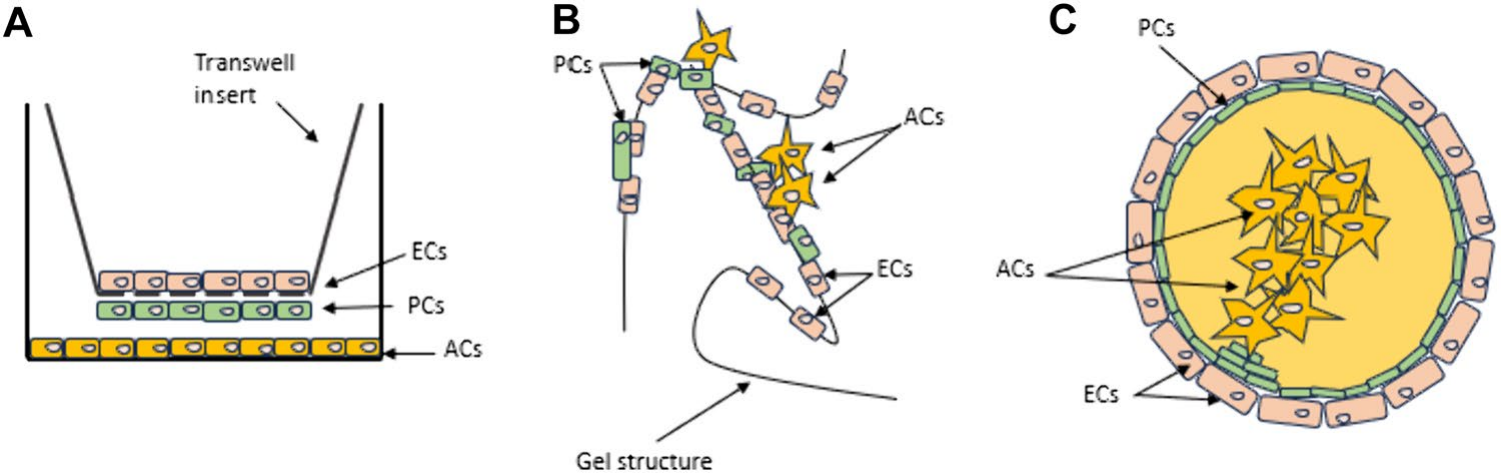
Transwell, Matrigel, and Spheroidal BBB model. In transwell model (**A**) ECs are grown on an insert to allow permeability studies between the two chambers. PCs are grown on the other side of the insert (to allow cell–cell interactions). ACs are grown in the bottom of the lower chamber (to allow the release of the factors into the medium). In Matrigel model (**B**) ECs form tube-like structures seeded in a gel structure. PCs adhere to ECs, while ACs are more loosely attached to the TJs. Spheroidal model (**C**) is independent of any type of scaffold. Cells self-assemble based on the intrinsic properties of each cell type (Urich et al. 2013)

The structural limitation of the 2D models led to the development of 3D BBB models (Aazmi et al. 2022). The transwell can be used for monolayer cell culture (Wu et al. 2021; Rice et al. 2022). This is currently the most commonly used method to study BBB. Co-culture of ECs, PCs, and ACs represents much better morphology and integrity of the barrier, when compared with monoculture of ECs or ECs cultured with PCs or ACs alone (Nakagawa et al. 2009). Particular cell lines can be seeded on both sides of the membrane with tiny pores to achieve contact co-culture. Transport mechanisms can be studied as the membrane separates luminal and abluminal parts (Fig. 2A). This one is cost-effective and fast. Few days are enough to obtain co-culture for functional testing, which make this model feasible for drug screening or genotyping. The transwell model can be used to determine endothelial integrity. Another possible use of transwell is: iPSC-derived cells is 3D culture with cells cultured in low-adherence conditions. Self-assembling into a tissue-like structure makes this model preferable to study neurotoxicity, disease modeling, and organogenesis. Culturing for several weeks allow to examine the long-term effects of drugs. However, the lack of shear-stress is the main disadvantage of transwell model and this type of co-culture system displays comparatively low levels of BBB regulatory proteins (Waldau 2019; Wu et al. 2021). Chung et al. have studied the effects of acute and chronic oxidative stress on the BBB in 2D and 3D models. BMECs derived from iPSCs cell line were used to assess the effect of H_2_O_2_ exposure on the BBB cells in transwell and 3D microvessels model. Exposition for 1 h represented the acute model, while over 10 days exposition was a chronic model. They hypothesized that tissue-engineered BMECs microvessels could enhance the response to oxidative stress and BBB model functionality (Chung et al. 2022).

### Matrigel Models

Tube-like human BBB model, based on hollow structures (channels) in 3D Matrigel, allows to obtain a platform with PCs and ACs on the abluminal side of the ECs (Urich et al. 2013). In Matrigel model ECs form tube-like structures seeded in a gel structure. PCs adhere to ECs, and ACs are more loosely attached to the ECs-PCs complex (Fig. 2B) (Urich et al. 2013). Endovascular progenitor cells can form tubular structures in Matrigel due to their self-assembly properties (Waldau 2019). Matrigel, gelatin, fibronectin, and laminin are possible culture substrates for neural cells (Komura et al. 2015). Matrigel is a gelatinous, high in various CNS proteins extracellular matrix mixture secreted by Engelbreth-Holm-Swarm mouse sarcoma cells (Berg et al. 2018; Wang et al. 2022). This substrate promotes the differentiation of various cell types (Kleinman and Martin 2005). Patel and Alahmad have studied the impact of different Matrigel sources on iPSCs differentiation into brain ECs. Matrigel provides growth factors and basement membrane for the maturation and differentiation of iPSCs into specific neural cells, allow to reconstruction of vasculogenesis. At the same time the source of Matrigel is of little consequence (Patel and Alahmad 2016). 3D Matrigel Model can be used to induce vascularized human brain organoids. This model has led to use 3D printing and bioprinting (Simöes Da Gama and Morin-Brureau 2022). Using the bioactive materials to introduce them into living cells allows to generate 3D model to study physiological mechanisms. 3D tissue models are very useful for infection experiments (Berg et al. 2018). A human BBB model with ECs, ACs, and PCs in 3D gel matrix mimics the permeability and gene expression profile observed in vivo. ECs derived from iPSCs were cultured with primary brain ACs and PCs to form the BBB microvascular networks (MVNs) (Waldau 2019). This type of material for the generation of organoids is not expensive and widely available but may be immunogenic. Furthermore, there is also poor control of mechanical properties of the scaffold (Kozlowski et al. 2021). Vascular permeabilities remain relatively close to the permeability in 2D models as well (Hajal et al. 2022).

### Spheroidal Model

BBB spheroids were established to study the transport of brain penetrating agents and organogenesis (Campisi et al. 2018). The use of iPSCs may allow to formation of patient-specific BBB models. ECs maintain their phenotype, cellular interactions, gene expression, vessel morphology, and functional barrier properties. In spheroidal models, ECs, ACs, and PCs auto-assemble without scaffolding material. ECs form the outer layer of the spheroid, when the PCs align as a monolayer on the surface and ACs form an astrocytic core. PCs separate the other two cell types (Fig. 2C) (Waldau 2019; Hajal et al. 2022). To build a spheroidal model, more neural cells should be used: microglia, oligodendrocytes, and neurons (Bhalerao et al. 2020). Neurons added to the culture increase the sensitivity to oxygen–glucose deprivation and better represent the interactions in the NVU (Stone et al. 2019).

In the spheroid model, cells are cultured in low-adherence conditions. Different cell types can be cultured together, so tissue-like structure can be obtained in the way of self-assembling. There are many possible applications of spheroidal models: to study organogenesis, neurotoxicity, cellular viral infectivity, permeability and function of different drugs, long-term effects of drugs, disease modelling. The integrity of spheroid can be tested only once, that can be considered as major disadvantage of the model, since transwell and tissue-on-chips models allow to perform of permeability tests many times (Wu et al. 2021).

**Fig. 3:**
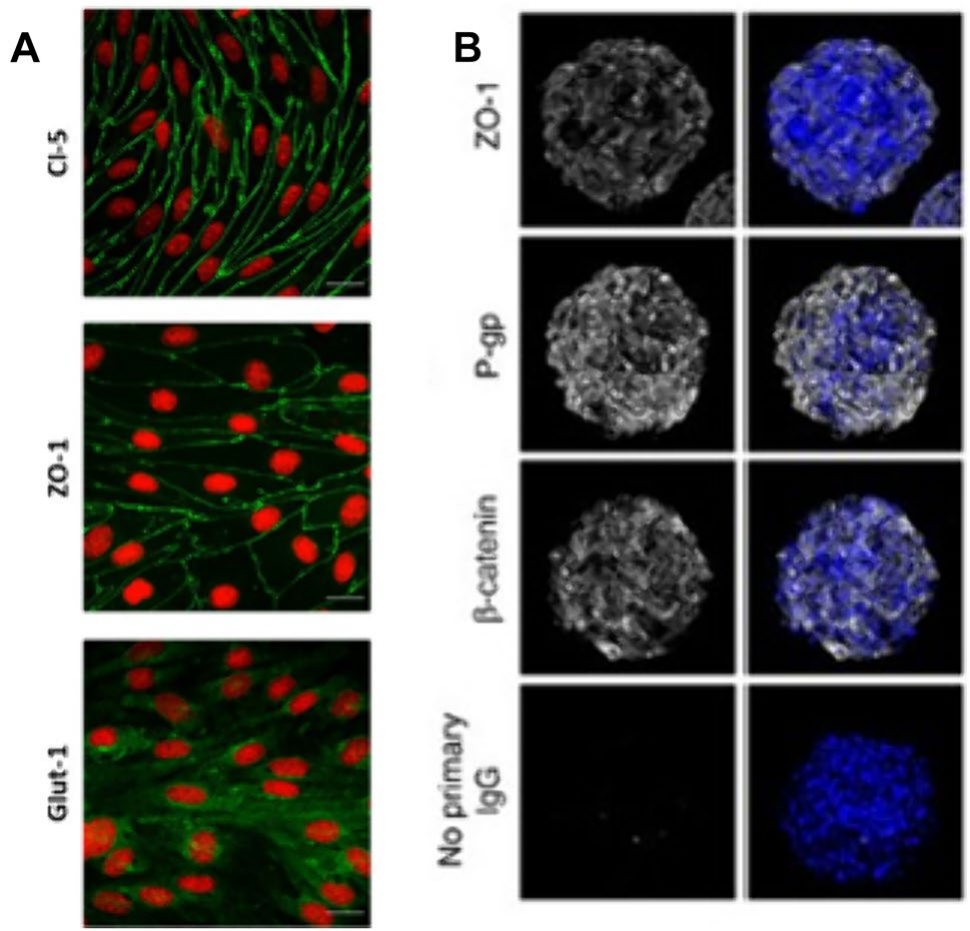
Identification of TJs and adherence junctions. ECs monolayer shows expression of claudin-5, ZO-1, and GLUT-1 (**A**); cell nuclei labeled with propidium iodide. Confocal images show expression of ZO-1 (TJ protein), β-catenin (adherens junctions), and P-gp (efflux pump) on the surface of BBB spheroid (**B**); cell nuclei labeled with Hoechst dye (shown in blue). Image A adapted from (Ozgür et al. 2022), image B adapted from (Cho et al. 2017)

Usage of the BBB spheroidal model is an appropriate tool to examine efflux of the ABCB1 substrates, including rhodamine123 and doxorubicin (Eilenberger et al. 2021). The spheroid surface exhibits a high expression of tight junction proteins. This 3D model allows imaging of tight-junction and transporter proteins, gene, and protein analysis (Hajal et al. 2022). Glycoprotein P (P-gp) and GLUT-1 proteins have a crucial role in the disposal of unwanted chemical compounds and the transport of glucose, respectively (Bhalerao et al. 2020). TJ proteins, claudin-5, and ZO-1 are a hallmark of BBB occurrence, confirming monolayer integrity (Fig. 3A) (Ozgür et al. 2022). Fluorescent cell labeling of spheroids enables identification of each cell type and cell interactions based on the presence of specific proteins: β-catenin, P-gp, and ZO-1 confirm 3D cellular organization and cell–cell contact, requisite for cell differentiation and barrier formation (Fig. 3B) (Cho et al. 2017). Knox et al. have shown that, the level of β-catenin, P-gp, and ZO-1 on the surface of spheroid is significantly higher than in the transwell model, even if ECs were co-cultured with PCs and ACs. Co-culture in transwell model have improved BBB tightness in comparison to 2D cell culture. Nevertheless, in the spheroidal model there is a direct cell–cell contact required for proper barrier formation (Nakagawa et al. 2009; Hatherell et al. 2011; Urich et al. 2013; Knox et al. 2022).

### Tissue-on-Chip

Tissue-on-a-chip approach uses microfluidic channels. A porous membrane is sealed among the channel networks, so cell populations can be introduced from both sides of the membrane and allowed to attach. The porous membrane among the cell culture chambers allows the migration of substances and interactions among different cell types similar to the transwell model. This technique has been used to establish many barrier models including the gut, lung, and vasculature. It is promising for drug delivery and CNS neurotoxicity studies (Kaisar et al. 2017). Wang et al. and Kilic et al. differentiated iPSCs into neuronal and astroglial cells. Wang et al. have developed brain organoids and confirmed that tissue-on-chip models allow to create microenvironment for brain organoids development, while Kilic et al. have studied cell migration due to gradients of chemotactic cues (Kilic et al. 2016; Wang et al. 2018). Tissue-on-Chip technique mimics an in vivo microenvironment, therefore tissues can be modeled more realistic. It enables to generate of shear stress through stimulated blood flow in BBB models (Wu et al. 2021). Microfluidic systems allow to control the 3D cellular and extracellular matrix; while they mimic cell-to-cell interactions and structures. They are referred as ‘tissue-on-a-chip’ (Campisi et al. 2018). The most used methods for the tissue-on-a-chip BBB model are live and dead cell imaging, permeability assays, immunofluorescence staining. qPCR is not very appropriable due to smaller cell amount than in other models (Wu et al. 2021). Moreover, currently available models using microfluidic channels still cannot precisely imitate in vivo BBB structure and function (Aazmi et al. 2022).

## Molecular Characteristic of BBB

BBB regulates the transition of substances between the blood and the cerebral parenchyma and, therefore, maintains the brain microenvironment (Fu et al. 2021). The barrier controls the transport of molecules, so neuronal function and chemical composition depend on the BBB permeability. NVU cells are involved in: the regulation of BBB permeability, oxygen delivery, neurotransmitter turnover, neurogenesis, and angiogenesis. Physiological BBB transport is based on molecular junctions of the barrier, endothelial, and pericyte transporters (Sweeney et al. 2019). TJs and adherent junctions (AJs) enhance the BBB separation function and limit the transcytoplasmic transport (Pandit et al. 2020).

**Fig. 4:**
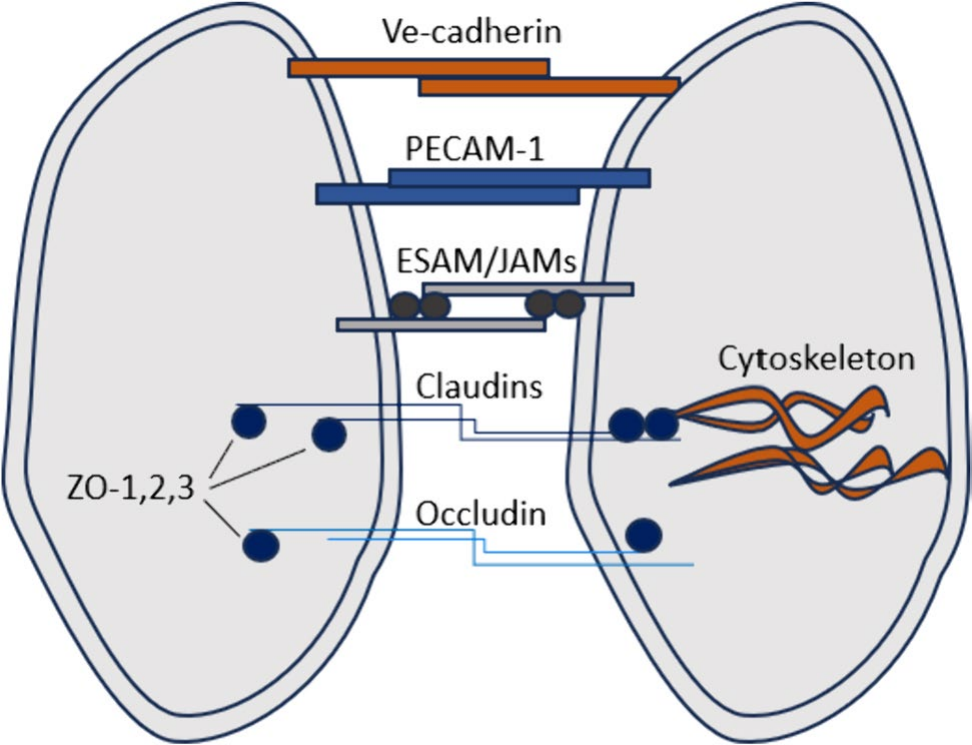
Brain endothelial connections. Ve-cadherin, PECAM-1, ESAM, JAM-A, -B, -C are junctional molecules that allow to maintaining tight sealing of the endothelial layer. Claudin-1, -3, -5, -12, and occludin limit solutes and ions crossing the barrier. ZO-1, -2, -3 together with claudins and occludins bind to cytoskeletal filaments to maintain the endothelial cytoskeletal network (Sweeney et al. 2019)

AJs proteins include: transcellular components, Ve-cadherin, platelet endothelial cell adhesion molecule-1 (PECAM-1), endothelial cell-selective adhesion molecule (ESAM), and JAMs (Knox et al. 2022). PECAM-1 and VE-cadherin are specific for endothelial cell-to-cell interactions (Vorbrodt and Dobrogowska 2003). ESAM, JAM-A, -B, and -C modulate junctional tightness similar to other AJs (Fig. 4) (Garrido-Urbani et al. 2014).

TJs are formed by the interaction among adjacent plasma membrane and integral transmembrane proteins (Bagchi et al. 2019). The TJ proteins include cadherins, catenins, claudins (claudin-1, -3, -5, -12), occludin, the membrane-associated guanylate kinase (MAGUK) protein family of zonula occludens (ZO-1, -2, -3) (Knox et al. 2022).

The BBB is enclosed by TJs (Tietz and Engelhardt 2015). Claudin-1, -3, -5, and -12 and occludin limit the ions paracellular transport and solutes across the BBB (Nitta et al. 2003). β-catenin stabilizes VE-cadherin and upregulates glucose transporter 1 (GLUT-1) and claudin-3 expression (once translocated into the nucleus) (Stenman et al. 2008). TJs are connected to the cytoskeleton via scaffolding proteins: ZO-1, -2, and -3 (Fig. 4) (Tornavaca et al. 2015). ZO-1 determines BBB tightness. Deficiency in claudins and ZO-1 is associated with BBB disruption and CNS diseases (Zlokovic 2011; Sweeney et al. 2018). Administration of TNF-like weak inducer of apoptosis (TWEAK) results in increased BBB permeability in mice due to decreases the level of ZO-1 in BMECs and increases the permeability of ECs monolayer (Wen et al. 2015).

ECs-PCs interactions are crucial for BBB formation and properties. Occludin, claudin-5, and ZO-1 expression can be decreased due to PCs deficiency (Bell et al. 2010). TJs and efflux transporters limit paracellular transport and control the entry of most therapeutic agents. Mouse-derived immortalized endothelial cell lines that present higher expression of claudin-5, occludins, and ZO-1 develop a tighter barrier. Loss of PCs can lead to the microvascular degeneration and BBB disruption. Additively, the actin cytoskeleton is supportive of the junctional proteins to anchor in the ECs (Fig. 4) (Knox et al. 2022).

## BBB Permeability

**Fig. 5:**
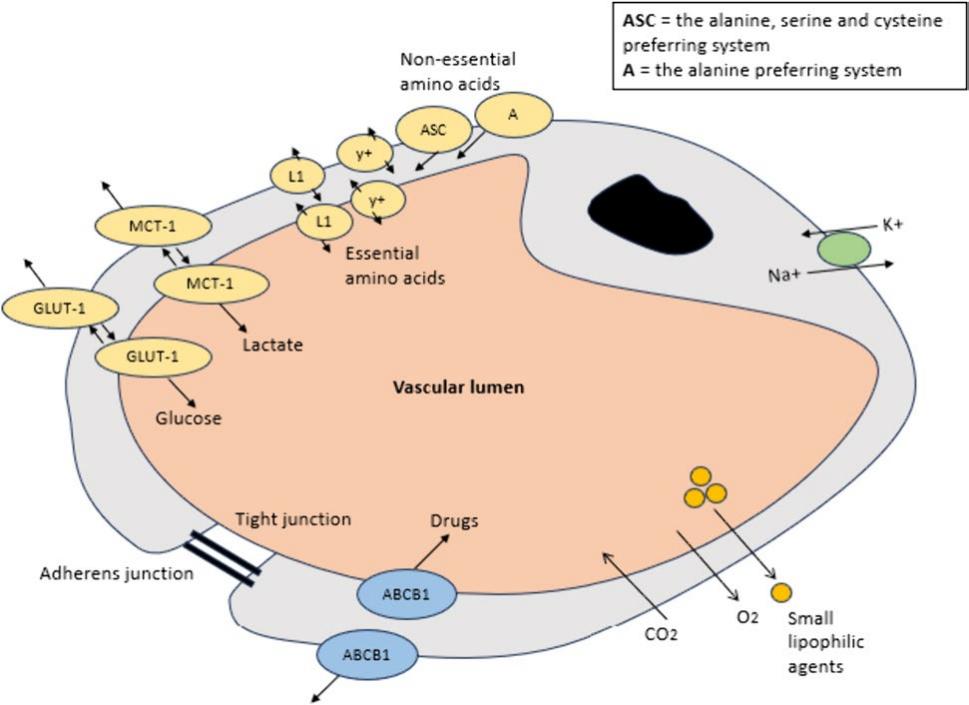
BBB transport mechanisms among the blood and endothelial cells. Oxygen, carbon dioxide, and small lipophilic drugs diffuse across the BBB. Ions require ATP-dependent transporters. GLUT-1, MCT-1, L1, and y + transporters transport nutrients, lactate, large neutral and cationic essential amino acids, respectively. Non-essential amino acid transporters are located at the BBB abluminal side and enable to remove glutamate or excitatory neurotransmitter that are neurotoxic from the brain (Zlokovic 2011)

Gases, e.g. carbon dioxide and oxygen, and small lipophilic molecules (< 400 Da) freely diffuse through the BBB, while the transport of other molecules is strictly regulated (Zhao et al. 2015). Substrate-specific transporters allow the distribution of amino acids, carbohydrates, fatty acids, nucleotides, hormones, amines, inorganic ions, or vitamins (Fig. 5) (Sweeney et al. 2019). Ions require transporters, such as ATPases (Zlokovic 2011). The brain has no system for energy storage; energy substrates are delivered to the brain and used directly after crossing the barrier (Sweeney et al. 2019). GLUT-1, monocarboxylate transporter 1 (MCT-1) for lactate transport, and transporters for large neutral and cationic essential amino acids are expressed on the both sides of BBB (Fig. 5) (Zlokovic 2011). GLUT-1, uniporter for glucose transport, is highly expressed in ECs (Winkler et al. 2015). The density of this transporter is significantly greater on the abluminal side of ECs that favor glucose transport from the blood into the brain (Simpson et al. 2001). It has a single binding site for glucose or other hexoses. While glucose concentration is lower in the brain in comparison to peripheral blood, GLUT-1 transports circulating glucose through the BBB. BMECs nutrient transporters facilitate the transport according to the concentration gradients (Zlokovic 2011; Deng et al. 2014). Haploid deficiency in GLUT-1 in murine ECs leads to TJ and basement membrane protein loss (Winkler et al. 2015). ATP-binding cassette (ABC) transporters, e.g. ABCB1 protein, are responsible for the active efflux of xenobiotics, drugs, and drug conjugates to prevent its accumulation in the brain. ABCB1 efflux Alzheimer’s Aβ toxin from the brain to the blood (Fig. 5) (Wang et al. 2016). Transporters for peptides, such as ABC proteins, include the endothelial receptor for natural anticoagulant, activated protein C (APC) (Fig. 5) (Guzman-Cottrill et al. 2008; Zlokovic 2011). Physiological activity of the BBB protects the CNS against any harmful substances as well as provides nutrients to the brain. However, on-demand BBB opening may help increase therapeutic agent penetration and improve treatment efficiency (Chen et al. 2022). Focused ultrasound (FUS) was used to increase BBB permeability temporarily. Except for affecting the tight packing of EC, astrocytes and miocytes are also activated by FUS (Chen et al. 2021). RBE4 were exposed to 12 MHz FUS for up to 30 min, which expanded intercellular spaces by remodeling the distribution of ZO-1. Nevertheless, FUS did not alter cell proliferation and oxidative marker, confirming the safety (Branca et al. 2023).

## BBB Dysfunction, Aging, and Neurodegeneration

The brain consumes ~ 20% and ~ 25% of body’s oxygen and glucose, respectively. When cerebral blood flow stops, neurons become to be damaged within minutes. Neurovascular disintegration, BBB and microvascular dysfunction and degeneration in the brain lead to neurodegenerative diseases. Deficiency in MVNs yields reduced brain nourishment and impaired clearance of neurotoxins. Vascular dysfunction is directly associated with neurodegeneration and neural impairment. Endothelial metabolic dysfunction, hypoperfusion, hypoxia, and BBB breakdown are the key pathways of vascular dysfunction and neurodegenerative disorders (Knox et al. 2022). In case of healthy aging, BBB integrity is impaired in the hippocampus, but with no cognitive disruption (Montagne et al. 2015). Increased IgG leakage into the brain, reduction of occludin, detachment of pericytes and decreased expression of endothelial genes induced by pericytes as well as decreased glycoprotein P (P-gp) expression and glucose uptake (due to change in expression of GLUT-1) constitute a hallmark of healthy aging (Erickson and Banks 2019; Yang et al. 2020; Knox et al. 2022).

BBB breakdown in disease is attributable to PCs detachment. Leakage of serum proteins and focal microhemorrhages lead to hemoglobin release, a source of iron, that catalyzes the formation of ROS. As a result, neurons are injured. The other representations of BBB breakdown are: altered paracellular and cellular transport, demyelination and neuronal damage, decreased TJ protein expression, detached and swollen astrocytes, pericytes loss and dysfunction, leukocyte infiltration, basement membrane thinning and activated microglia (Knox et al. 2022). Hypoperfusion, a reduced cerebral blood flow, and hypoxia promoted by vasogenic edema exacerbate neuronal damage. The result of these processes is diminished ATPases activity and ATP synthesis, altered pH and electrolyte balance, and consequently accumulation of neurotoxins and glutamate in the brain (Kalaria 2010; Moskowitz et al. 2010). Hypoperfusion affects protein synthesis and therefore synaptic plasticity (Iadecola 2004). Neurotoxic proteins (e.g. plasmin, fibrin, and thrombin) can enter the brain, when BBB is untight (Zlokovic 2011). Neuronal laminin is degraded by accumulated plasmin that promotes neuronal injury (Chen and Strickland 1997). The level of TJ and AJ proteins decreased in neurodegenerative disorders like AD or multiple sclerosis (Bell et al. 2010; Zlokovic 2011). These neurological conditions cause leukocytes leakage into the brain and loss of zonula occludens and occludin (Ballabh et al. 2004).

## BBB Under Hypoxia

**Fig. 6:**
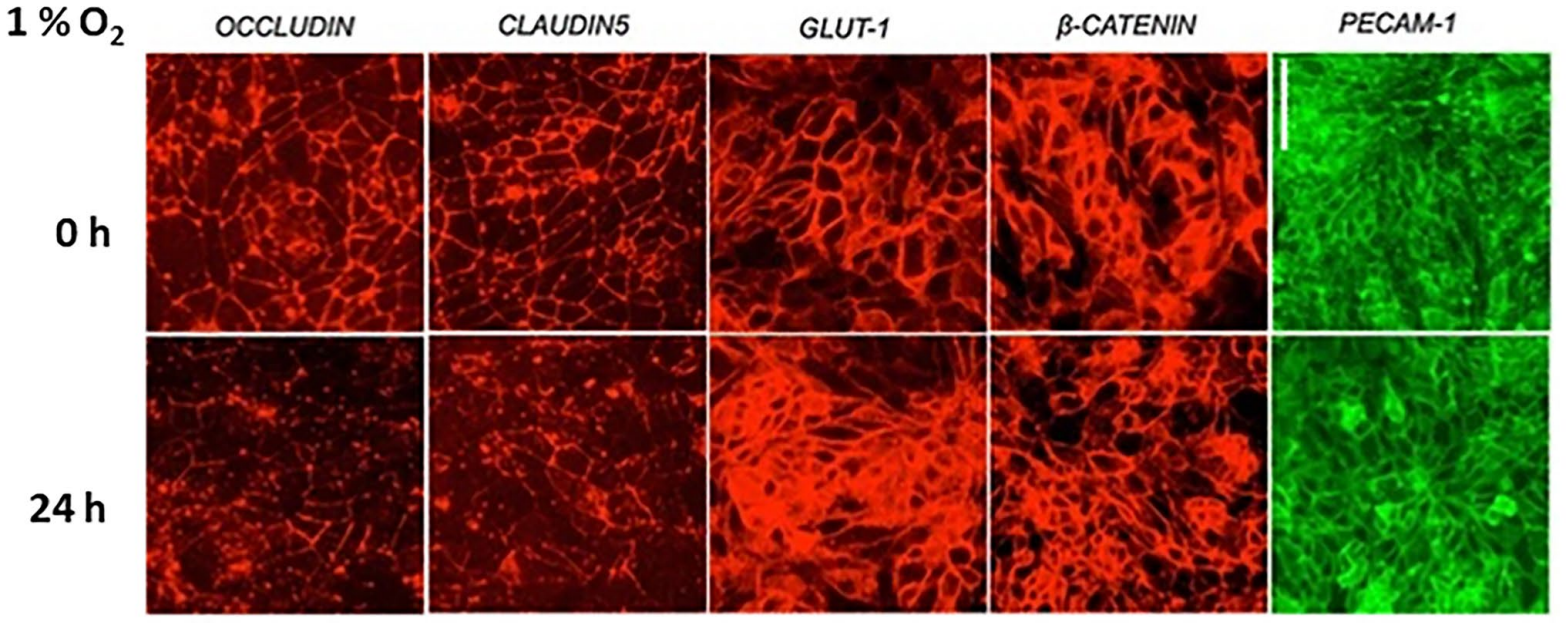
TJs immunostaining (Occludin, Claudin-5) and AJs (GLUT-1, β-catenin, PECAM-1) proteins in BMEC monolayer exposed to 24 h hypoxia (1% O_2_); showing changes in the TJs protein expression related to O_2_ deprivation, while AJs remained unaffected. Scale bare 20 µm. Image adapted from (Page et al. 2016)

Hypoxia in the brain leads to its damage and BBB breakdown. It could be attributed to strokes or neurologic diseases. Low-oxygen concentration influences the expression levels of TJs (Brown and Davis 2005; Nzou et al. 2020) in BBB, efflux transporters, solute carriers, and receptors for nutrients and hormones. Hypoxia-induced cell response is HIF-1α mediated (Lee et al. 2012; Engelhardt et al. 2014) and it is cell-specific. ECs are more sensitive to hypoxia than ACs and PCs (Engelhardt et al. 2015). Moreover, ACs and PCs represent a synergistic effect in barrier improvement, when co-cultured with RBE4 under O_2_ deprivation (Hayashi et al. 2004; Al Ahmad et al. 2009). Engelhardt et al. reported that GLUT-1 (Yamagata et al. 2004) and VEGF expression in the ECs under O_2_ deprivation was increased, when compared to the ACs and PCs (Engelhardt et al. 2015). Also, in vivo study on mice showed increased levels of VEGF mRNA and protein. VEGF is an angiogenic growth factor, thus inducing new vessel formation leading to enhanced vascular permeability (Schoch et al. 2002). Yeh et al. demonstrated VEGF expression regulation by the HIF-1α. They used a rat animal model to inhibit HIF-1α by 3-(5’-hydroxymethyl-2’-furyl)-1-benzylindazole, leading to decreased VEGF production and cause the BBB protection (Yeh et al. 2007). Hypoxia-induced changes in the stem cell-derived human BMECs and immortalized BBB cell line were verified by Page et al. The TJs complexes in both cell lines were disrupted (Fischer et al. 2002; Al Ahmad et al. 2009). However, AJs remain unaffected (Fig. 6) (Page et al. 2016). Claudin-5 expression was analyzed in the monolayer culture of bEND.3. Within the barrier formation by bEND.3 cells, claudin-5 was relocated from the cytoplasm to the plasma membrane, creating TJ. While cells were exposed to hypoxia, claudin-5 expression was decreased (Koto et al. 2007). RBE4 exposed to hypoxia showed increased ROS formation providing EC disintegration. This phenomenon led Ahmad et al. to inhibit ROS generation by diphenyliodiunium and verify, whether it leads to maintaining BBB function, demonstrating promising results of using ROS inhibitors for supporting BBB function during stroke or cerebrovascular injury (Al Ahmad et al. 2012).

However, in this review, the disruptive effect of hypoxia on BBB is widely discussed. Ozgür et al. reported that functional BBB with tight monolayer of bovine brain capillary endothelial cells (BCECs) was present during the early development of the brain vasculature, when the oxygen concentration was low. The expression level of the transport proteins, such as GLUT-1 and P-gp was increased under hypoxia (Park et al. 2018), which was confirmed by the increased glucose uptake. Furthermore, BBB tightening was observed when confluent cells were exposed to hypoxia (Ozgür et al. 2022).

## BBB Under Neurotoxic Condition

Many chemicals, such as heavy metals or pesticides are neurotoxic. The toxic effect on the brain depends on the dose, brain development, and mode of action (Pistollato et al. 2020). Moreover, when pregnant women, newborns, or young people are exposed to neurotoxins, the normal development and maturation of the nervous system can be disturbed (Parran et al. 2005). Metals are essential for proper CNS functioning, however when their optimal level is exceeded, or heavy metals cross the BBB barrier, leakage occurs, especially when the barrier is immature (Lewis and Zheng 2007). Similar to hypoxia, the response to the metals is cell-depended. Usually, ACs became the target of metal toxicity (Li et al. 2021). However, lead (Pb) rather accumulates in ECs than in another BBB cells (Zheng et al. 2003). In vivo study on rats showed that neurotoxins, such as Pb, increase BBB permeability due downregulation of TJ proteins, ZO-1, and occludin. Interestingly, when a low and high dose of Pb was compared, a significant difference in protein expression was noticed (Struzyńska et al. 1997). Low Pb dosage decreased the expression of occludin, while the level of ZO-1 was unaffected. This indicates that ZO-1 is less sensitive to Pb exposure (Song et al. 2014). Mercury (Hg) provoke BBB leakage, however the level of toxicity, thus barrier breakdown depends on Hg compound. Inorganic Hg is less toxic than organic ones, for example, methylmercury (MeHg). Highly lipophilic MeHg can easily diffuse through the cell membrane without any carrier proteins (Zheng et al. 2003). Rats had been exposed to the MeHg, which induced BBB damage due to the upregulation of VEGF expression. This phenomenon was predominant in astrocytes (Takahashi et al. 2017). Hirooka et al. observed similar dependence within the human brain microvascular endothelial cells and PCs, where the MeHg increased VEGF expression and therefore provided barrier leakage (Hirooka et al. 2013). Except for the disruptive effect of metals, it was shown that zinc (Zn) may have beneficial effect by blocing the action of cadmium, thus preventing ZO-1 downregulation and dislocation (Branca et al. 2022). Interestingly, barrier disarrangement by heavy metals could be treated with chelation therapies (Ferrero 2022).

**Fig. 7:**
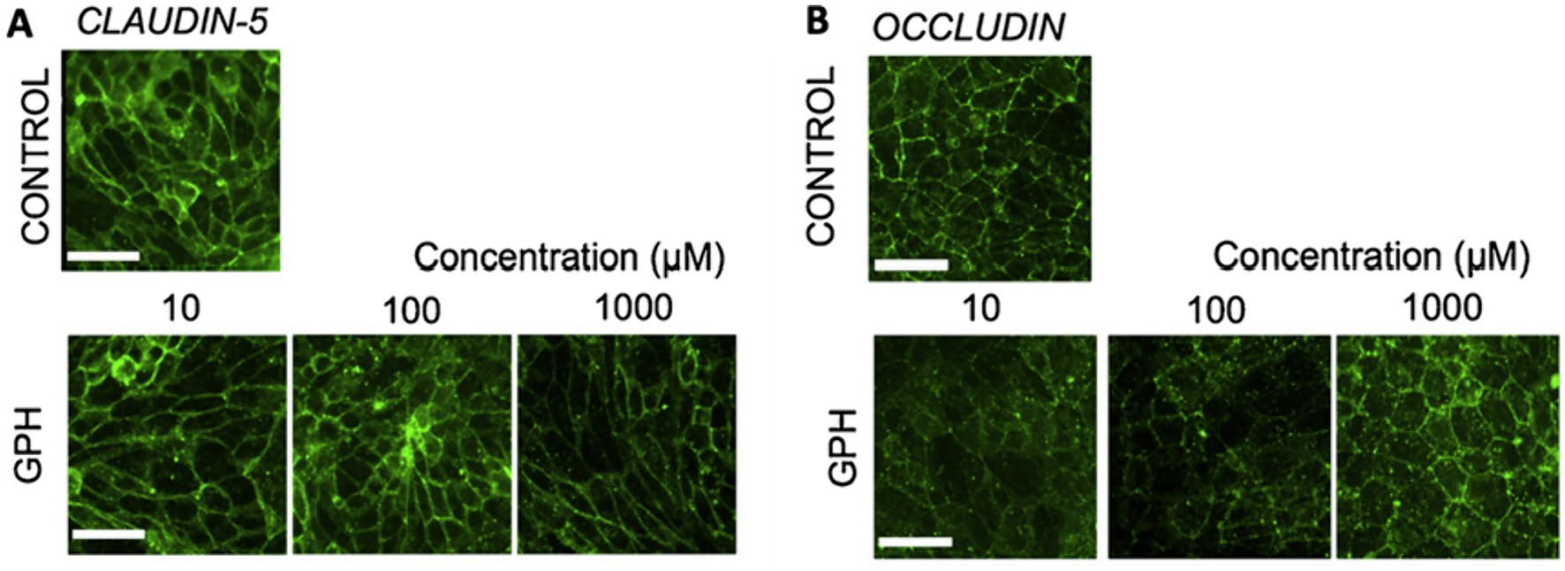
Immunostaining of (**A**) Claudin-5 and (**B**) Occludin (TJs proteins) in BMEC monolayer exposed to GPH, showing decreased protein level within an increasing concentration of GPH. Scale bare 50 µm. Image adapted from (Martinez and Al-Ahmad 2019)

Parran et al. analyzed the effect of pesticide—chlorpyrifos on BBB formed by co-culture of BMEC with rat astrocytes, showing increased barrier permeability (Parran et al. 2005). Moreover, the integrity of BBB built from the same cells as described above was verified with another pesticide—malathion; again, showing decreased tightness (Balbuena et al. 2010). The monolayer of iPSC-derived BMECs was treated with glyphosate (GPH)—a herbicide, to assess the BBB permeability and TJs proteins. The glucose uptake increased, as the GLUT-1 expression was greater than in the untreated cells. Furthermore, both claudin-5 (Fig. 7A) and occludin (Fig. 7B) expression were downregulated, confirming BBB breakdown. Here, the toxic effect of metals and pesticides on BBB was described, as those two groups of compounds are the most frequent and least examined recently (Martinez and Al-Ahmad 2019).

## Conclusions and Perspectives

Restoring the disrupted BBB may decelerate the progression of the neurodegenerative diseases. Tightening the barrier could restrict the negative effect of the inflammation. In vitro BBB models are useful tools to study BBB physiology and molecular mechanisms that occur in the barrier. In vivo models are used for drug screenings and safety and efficacy assessment, while clinical models are useful for studying BBB function and disruption in diseases (Knox et al. 2022).

BBB models are essential for neurodegenerative disease studies and drug discoveries. At the beginning 2D models were used, then that 3D systems were developed to better mimic neural conditions (Mantecón-Oria et al. 2022). Functional tests performed on 2D models were not enough credible to use them as a reference model for BBB studies (Buzhdygan et al. 2020; Hajal et al. 2022). The simplest 3D model, transwell, is cost-effective and fast, nevertheless, the lack of shear-stress and comparatively low levels of BBB regulatory proteins constitute the main disadvantages of this model (Waldau 2019; Wu et al. 2021). Moving forward to Matrigel based models, in which ECs form tube-like structures, we are becoming closer to the system for physiological mechanisms studies. This approach is useful for gene expression profile assessment, infectious experiments. Nevertheless, may be immunogenic and vascular permeabilities are still far away from in vivo conditions (Berg et al. 2018; Hajal et al. 2022; Simöes Da Gama and Morin-Brureau 2022). Therefore spheroidal model, auto-assembled without scaffolding material, has been developed. Spheroids allow to study of cellular interactions, gene expression, vessel morphology, and functional barrier properties. The limitation is the fact that spheroid can be tested only once as it disintegrate very easily (Waldau 2019; Wu et al. 2021; Hajal et al. 2022). The last one model discussed in this review, tissue-on-a-chip, uses microfluidic channels and is appropriate for drug delivery and CNS neurotoxicity studies. The ability to generate shear stress through stimulated blood flow in BBB models makes this technique very promising (Kaisar et al. 2017; Campisi et al. 2018; Wu et al. 2021).

Many treatments fail at the screening stage due to inability to cross the BBB; platforms that better mimic the functionality of the human barrier are needed. Current models that facilitate cellular interactions are still far from in vivo BBB, therefore the development of better 3D models, the microfluidics, and 4D biofabrication will be the future of BBB in vitro modelling (Mantecón-Oria et al. 2022). Combination of patient-derived iPSCs with Organ-Chip technology can serve to provide a platform for modelling disorders, drug discovery, and personalized medicine (Vatine et al. 2019).

## Data Availability

Data sharing is not applicable to this article as no new data were created or analyzed in this study.

## Abbreviations

ABC: ATP-binding cassette
ACs: Astrocytes
AD: Alzheimer’s disease
AJ: Adherent junction
APC: Activated protein C
BBB: Blood brain barrier
BCECs: Bovine brain capillary endothelial cells
bEND.3: Mouse brain endothelial cells
BMECs: Brain microvascular endothelial cells
CNS: Central nervous system
CPS: Chlorpyrifos
ECM: Extracellular matrix
ECs: Endothelial cells
ESAM: Endothelial cell-selective adhesion molecule
FUS: Focused ultrasound
GLUT-1: Glucose transporter 1
hCOs: Human cortical organoids
hESCs: Human embryonic stem cells
iPSCs: Induced pluripotent stem cells
JAM: Junctional adhesion molecules
MAGUK: Membrane-associated guanylate kinase
MCT-1: Monocarboxylate transporter 1
MVNs: Microvascular networks
NVU: Neurovascular units
PCs: Pericytes
PECAM-1: Platelet endothelial cell adhesion molecule-1
P-gp: Glycoprotein P
RBE4: Rat brain endothelial cells
ROS: Reactive oxygen species
TJ: Tight junctions
TWEAK: TNF-like weak inducer of apoptosis
VEGF: Vascular endothelial growth factor
ZOs: Zonula occludens
